# Cognitive aging at work and in daily life—a narrative review on challenges due to age-related changes in central cognitive functions

**DOI:** 10.3389/fpsyg.2023.1232344

**Published:** 2023-08-09

**Authors:** Stephan Getzmann, Julian E. Reiser, Patrick D. Gajewski, Daniel Schneider, Melanie Karthaus, Edmund Wascher

**Affiliations:** Leibniz Research Center for Working Environment and Human Factors at the Technical University of Dortmund (IfADo), Dortmund, Germany

**Keywords:** executive functions, cognitive control, aging, daily life, work-related activities, EEG

## Abstract

Demographic change is leading to an increasing proportion of older employees in the labor market. At the same time, work activities are becoming more and more complex and require a high degree of flexibility, adaptability, and cognitive performance. Cognitive control mechanism, which is subject to age-related changes and is important in numerous everyday and work activities, plays a special role. Executive functions with its core functions updating, shifting, and inhibition comprises cognitive control mechanisms that serve to plan, coordinate, and achieve higher-level goals especially in inexperienced and conflicting actions. In this review, influences of age-related changes in cognitive control are demonstrated with reference to work and real-life activities, in which the selection of an information or response in the presence of competing but task-irrelevant stimuli or responses is particularly required. These activities comprise the understanding of spoken language under difficult listening conditions, dual-task walking, car driving in critical traffic situations, and coping with work interruptions. Mechanisms for compensating age-related limitations in cognitive control and their neurophysiological correlates are discussed with a focus on EEG measures. The examples illustrate how to access influences of age and cognitive control on and in everyday and work activities, focusing on its functional role for the work ability and well-being of older people.

## 1. Introduction

Demographic change is affecting most Western countries and leading to an increase in the proportion of older people in society (European Commission, 2018) and also on the labor market, a development referred to as “workforce aging” (Aiyar and Ebeke, 2016). Combined with an overall increase in life expectancy and the associated strain on social security systems, there are growing calls in many countries for working lives to be extended. This is accompanied by an increasing shortage of skilled workers, which poses major challenges for the economy in Europe and other countries (Brunello and Wruuck, 2021). At the same time, work itself, work activities and the associated demands on working people have changed significantly in recent decades (Young et al., 2015). For example, the digitization of work activities, the acceleration of work processes, but also the expansion of the service sector are placing increasingly higher demands on the cognitive performance of employees. These include, above all, the ability to adapt quickly to changing work requirements, the continuous acquisition of new skills and the use of new technologies, as well as a high capacity to process information while maintaining a high level of mental flexibility. This places demands on various mental functions of workers, which, however, are often subject to age-related changes (e.g., Wolf et al., 2018). To put it pointedly, specific work requirements will in future increasingly encounter an employee structure with a reduced ability to adapt to these very requirements. This may result in older employees being overstrained and—as a consequence—in health hazards and the risk of premature retirement from working life.

It therefore seems necessary to better understand the interplay of situational demands, the cognitive functions required for them, and their changes with age. Here, we illustrate these interrelations by means of four selected activities and actions, all of which are of high, if not prototypical, importance for our everyday life and work. The focus in each case is on cognitive control, a mental function that plays a special role in all four areas, and on its neuro-physiological foundations. After a brief introduction to the topic of cognitive control, work, and aging, recent work will be reviewed in which specific functions of cognitive control have been investigated in close-to-reality everyday and work-related experimental settings. A special focus is on neuro-cognitive correlates of these processes and how they can be mapped using neurophysiological methods.

## 2. Executive functions and cognitive control: theoretical models, neuro-cognitive correlates, and effects of age

Executive functions (EF) optimize goal-directed behavior and counters automaticity. Cognitive control is a term usually associated with the functioning of the prefrontal cortex PFC and associated regions such as the cingulate cortex (Friedman and Robbins, 2022) underlying specific executive functions (Diamond, 2013). Cognitive control involves mental mechanisms that allow the coordination and configuration of sensory, cognitive, and motor systems to serve the achievement of higher-level goals (for review, Gratton et al., 2018). Cognitive control comes into play whenever goal achievement is complicated or compromised by a new (and therefore often untrained) or conflicting constellation of stimuli and/or responses. Goschke (2017) described several examples of when cognitive control is needed. These primarily include situations where (a) in a complex planning process (such as writing this review), distinct subgoals and their temporal sequence need to be specified, (b) competing and potentially distracting stimuli as well as inadequate habitual responses or competing motivational tendencies need to be suppressed, (c) new or unpracticed actions need to be performed, or (d) high flexibility is required when switching between goals or tasks. All these processes play significant roles in mental activities in everyday life as well as in professional life and are important for safe, efficient and healthy work. However, experimental research so far uses paradigms that mostly involve abstract and less holistic tasks. Gratton et al. (2018) described three different groups of paradigms that are widely used to assess EF. These comprise updating tasks (testing a person’s ability to continuously update information in working memory such as in the N-back task), shifting tasks (testing the ability to switch between different stimulus–response pairings such as in task-switching paradigms), and inhibition tasks (testing the ability to suppress a prepotent or prepared response such as in a Go/Nogo task). It has been shown that these core EF—although moderately correlating with each other—contribute differentially to task performance (Miyake et al., 2000). Cognitive control is also required to detect and reduce errors and to enhance learning (Holroyd and Coles, 2002).

With increasing age, changes occur in almost all areas of human cognitive functions (for review, Craik and Salthouse, 2011). In addition to frequently described changes in long-term memory functions such as episodic memory (for review, Naveh-Benjamin and Mayr, 2018), cognitive aging concerns changes in working memory functions, in the speed with which information is processed and in EF (e.g., Lustig and Jantz, 2015; Zanto and Gazzaley, 2017). These are higher-level cognitive skills that allow us to coordinate and control other cognitive processes to enable goal-directed behavior (Diamond, 2013), including problem solving, abstract thinking, and emotion regulation. Impaired cognitive control is especially critical because it monitors and controls other mental functions, such as inhibitor or attentional processes and the use of working memory. A study on the development of EF over the lifespan showed clear differences in the trajectories (Ferguson et al., 2021). While inhibitory control, working memory and planning continuously decreased starting in middle age, measures of cognitive flexibility showed different developments, with switch costs decreasing and mixing costs increasing. However, influences of such changes on cognitive performance are sometimes difficult to detect by means of experimental behavioral paradigms, and sometimes lead to contradictory findings. A meta-analysis of the results of 176 studies on inhibition in old age revealed deficits only in very few tasks like Go/Nogo and stop-signal tasks, but not in most tasks (Rey-Mermet and Gade, 2018), which calls into question the general validity of the inhibition deficit hypothesis (Hasher and Zacks, 1988), assuming that there is a general inhibition deficit in older age. Furthermore, decreases in cognitive control interact in a complex way with compensatory strategies, which may complicate interpretation of behavioral outcomes (Lustig and Jantz, 2015). Therefore, it seems necessary to consider the neurophysiological basis of age-related changes in EF and to include neurophysiological measures such as EEG or functional magnetic resonance imaging (fMRI).

Here, the prefrontal cortex (PFC) plays a special role, which is central for EF and several other cognitive functions (Menon and D’Esposito, 2022), and for which a number of age-related changes have been demonstrated (for review, Zanto and Gazzaley, 2019). According to the frontal aging hypothesis (initially proposed by West, 1996) the majority of age-related cognitive changes are therefore based on reduced efficiency of the frontal lobes. For example, structural changes such as a decrease in PFC volume with age were observed (Maillet and Rajah, 2013), with larger PFC volume and greater PFC thickness being associated with better executive functioning (Yuan and Raz, 2014). These age effects include not only a decrease in cortical activity, but also changes in the activity pattern. For example, the hemispheric asymmetry reduction in older adults (HAROLD) model assumes that prefrontal activity during cognitive tasks are less lateralized in older adults than in younger adults (Cabeza, 2002; Cabeza et al., 2002), while the PASA (posterior–anterior shift) model assumes a shift in activity in posterior regions toward more anterior regions with increasing age (Dennis and Cabeza, 2011). Both models are supported by functional neuroimaging and behavioral evidence, and have been interpreted as correlates of a global reorganization of neurocognitive networks as well as regional neural changes (for review, Eyler et al., 2011). In particular, bilateral and more frontal activity in older adults may reflect dedifferentiation and compensatory processes for age-related deterioration through recruitment of fronto-centrally localized EF. In a similar, albeit more comprehensive direction, is the compensation-related utilization of neural circuits (CRUNCH) hypothesis, assuming that additional brain regions are activated in older age to compensate for declining brain functions when performing cognitive tasks (for review, Kang et al., 2022). More specifically, it is assumed that older adults activate more cognitive resources even at lower task demands, as shown for example by fMRI studies demonstrating compensatory over-activation compared to younger adults (e.g., Reuter-Lorenz and Cappell, 2008). Finally, the scaffolding theory of aging and cognition (STAC) theory, provides an adaptive model of neural aging, in which the development of complementary structures and functions are part of a lifespan process to preserve cognitive functioning despite cognitive declines. The STAC model aims at the interactions of adverse and compensatory neural processes to explain the variance in cognitive performance and therefore refers to results from structural and functional neuroimaging (for review, Festini et al., 2018).

The examination of assumptions within these theoretical frameworks have benefited in the past (and still do) from neuropsychological approaches such as lesion studies as well as from neurophysiological methods targeting the neuro-cognitive correlates of cognitive control. Cognitive control can be assessed by various neurophysiological measures such as fMRI and functional near-infrared spectroscopy (fNIRS). The present review focuses on the EEG signal, from which several measures can be derived that indicate increased cognitive control, for example, as a reaction to the handling of a demanding mental task. One such measure is the oscillatory activity over fronto-central brain areas, especially activity in the theta frequency band (approximately 3–8 Hz; Cavanagh and Frank, 2014), that has even been regarded as a common mid-frontal substrate for cognitive control processes (Cavanagh et al., 2012). Numerous studies showed that high fronto-central theta activity is related to high mental processing demands and associated with higher cognitive workload (Gevins et al., 1997; Borghini et al., 2014; for a meta-analysis, see Chikhi et al., 2022) or task engagement (Onton et al., 2005). A commonly found, albeit indirect, evidence of cognitive control is a reduction in activity measured over posterior areas in the alpha frequency band (8–12 Hz). High alpha activity is associated with states of mental withdrawal and inattention, such as daydreaming and mind wandering (Compton et al., 2019; Arnau et al., 2020). A reduction in alpha activity in response to a stimulus to be processed is therefore often associated with mental resource deployment (Wascher et al., 2016) and attentional engagement (Pattyn et al., 2008) as a consequence of increased cognitive control (for review, Klimesch, 1999; for a recent review on the role of alpha activity for attentional control, Schneider et al., 2022).

Considering the assumption of age-related changes in cognitive control, it seems plausible to find evidence for changes in these electrophysiological correlates as well. However, the evidence is sometimes ambiguous and even contradictory. While earlier work often assumed a decline in overall cortical activity with age (e.g., Salthouse, 1988), later work showed a decline in activity in the alpha and theta frequency bands (e.g., Cummins and Finnigan, 2007; Nowak et al., 2021; Tröndle et al., 2023), while other work showed no changes in alpha activity (Caplan et al., 2015) and rather increased theta activity (Klass and Brenner, 1995; Ishii et al., 2017). Especially in older adults, increased fronto-central theta activity has been associated with better performance in tasks strongly associated with cognitive control (Finnigan and Robertson, 2011; Vlahou et al., 2014). In line with the CRUNCH model, this could indicate allocation of extra mental resources to compensate for declining brain functions (which may be indicated by a decreased alpha activity) when performing demanding cognitive tasks. However, a recent analysis of a larger number of older subjects aged 60–80 years demonstrates a decrease in individual alpha peak frequency (IAF), but no correlation of age and oscillatory power in the alpha and theta bands (Cesnaite et al., 2023).

In addition to induced changes in brain oscillatory activity, event-related potentials (ERPs) evoked by synchronized postsynaptic neural activity are important correlates of cognitive control. Especially the N2 component, which occurs over frontocentral brain areas about 200–400 ms after a stimulus event, should be mentioned here (Folstein and Van Petten, 2008). The interpretation of the significance of the N2 depends to some extent on the experimental paradigm in which it is measured, as is the case with almost all ERP components. In general, however, the N2 is associated with cognitive control in conflictual situations and with conflict monitoring processes when inhibitory control is needed (Larson et al., 2014). In this context, it is also worth mentioning the P3b, a robust component that typically occurs 300–600 ms after a significant stimulus event over posterior brain areas. This is associated with the provision of mental processing resources, context updating (Polich, 2007), and response selection (Verleger, 2020).

Regarding age-related changes in ERP correlates of cognitive control, there are mixed findings with respect to N2 amplitude. A reduction in N2 amplitude and increase in N2 latency with increasing (older) age is often found (for reviews, see Friedman, 2012; Hämmerer et al., 2014; Pires et al., 2014), for example in auditory oddball (Anderer et al., 1996), task switching (Gajewski et al., 2018), and working memory (Nowak et al., 2021) tasks, as well as a meta-analysis on Go/Nogo tasks (Cheng et al., 2019). While this is in line with the inhibition deficit hypothesis (Hasher and Zacks, 1988), other papers found no changes in N2 in adulthood, for example in a visual flanker task (Reuter et al., 2019). Finally, also increases in N2 have been reported, which—in combination with a decrease in P3b—could be related to the above-mentioned PASA model and a compensatory activation of prefrontal cortical areas (Kropotov et al., 2016), according to the CRUNCH model. There is much evidence for age-specific changes in the P3b, represented by reduced amplitude and increased latency with age (for review, Friedman, 2012), which has been related to a decrease and slower allocation of processing resources at older ages. Overall, it can be stated that the findings of age-related changes in EEG activity seem to strongly depend on situational factors and the investigative approach used. Accordingly, a recent review did not indicate a strong association of neurophysiological markers of age-related changes and behavioral effects, arguing for a “qualitative (instead of or along with a quantitative) difference in the deployment of cognitive resources in aging” (Tagliabue and Mazza, 2021, p. 1). Experimental paradigms that are more closely related to the tasks and challenges of older people in everyday life might be relevant here.

## 3. Effects of age-related changes: examples from everyday life and work

**Figure 1:**
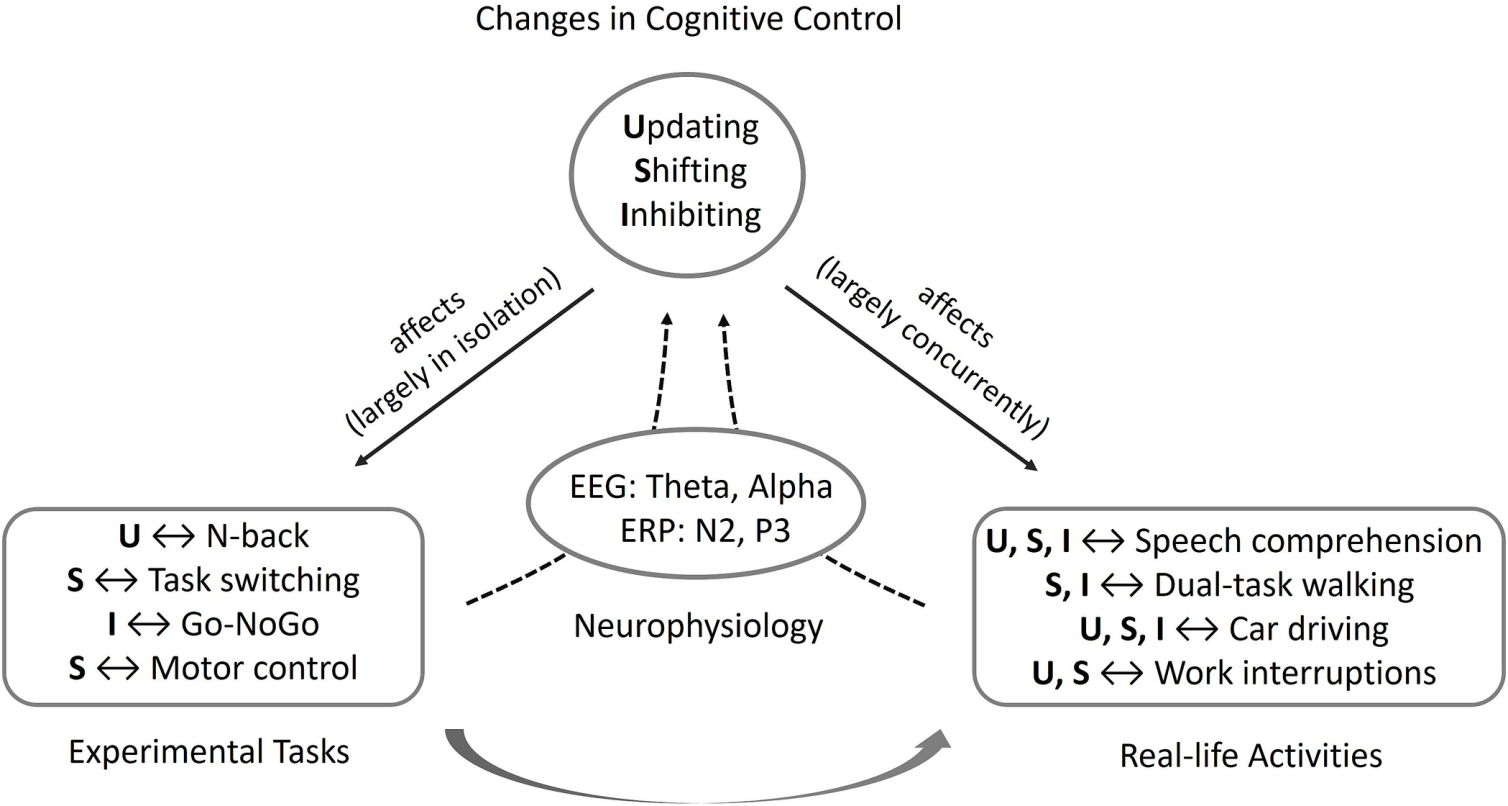
Conceptual representation of the relationship between cognitive control, its influence on (and capture by) experimental paradigms and everyday activities, and its mapping by electrophysiological correlates. While classical experimental tasks ideally target the core processes of cognitive control updating (U), shifting (S), and inhibition (I) in isolation, in everyday activities, several processes usually play a role at the same time.

In view of the importance of intact EF for everyday life and work, the question arises how age-related changes in EF can be investigated in everyday and work-related settings. Therefore, research paradigms seem to be necessary that focus on everyday tasks and challenges of older people. In addition, it seems important to evaluate which possibilities exist to compensate for these changes in order to protect the older person from excessive demands on the one hand, and to ensure safety and efficiency in everyday life and at work on the other. In the following, mechanisms of cognitive control and their neurophysiological correlates are illustrated on the basis of four prototypical examples in which inhibition, shifting, and updating processes as well as age-related changes play an important role for everyday life and work activities (Figure 1). These focus on the protection against competing and potentially distracting stimuli in favor of relevant information such as in speech comprehension under difficult listening conditions, the motor-cognitive coordination needed while dual-task walking, the rapid adaptation of attention to changing conditions and situations as needed when driving a car in critical traffic situations, and the flexible control of working memory processes when coping with work interruptions.

### 3.1. Speech comprehension

Verbal communication is of utmost importance in daily life and in many areas of professional life. Examples include a conversation with friends in a busy restaurant or a teacher’s job, where verbal communication is essential and where speech information must be attended to and responded to in an environment with background noise. Unless there are serious impairments of hearing, understanding spoken language is an automatic process that makes no special demands on cognition, whereas speech comprehension under complex conditions, i.e., in the presence of background noise and competing speakers, involves considerable effort (for review, Bronkhorst, 2015). Such conditions are referred to as “cocktail party scenarios” (Cherry, 1953). In order to perceive relevant speech information, an auditory scene analysis and the segregation of relevant and irrelevant (speech) information is necessary (Bregman, 1990). Here, attentional processes play an essential role, ensuring that (a) after successful segregation the focus of auditory attention is (and remains) on the relevant speaker and that (b) the relevant speech content is protected from competing content and noise. In this context, cognitive control plays an important role, especially when the processes of scene analysis, speaker segregation, and attentional focusing have to be performed repeatedly and rapidly in a changing speaker environment in order to follow a conversation between several talkers (e.g., Wächtler et al., 2022).

Age is typically associated with impairment in these processes. There is a combination of increasing deterioration in basal auditory performance, language-specific processes, and higher cognitive functions (Humes and Dubno, 2010). While the first two processes make the understanding of spoken language more difficult, changes in cognitive control lead to an easier distractibility by noise and increased difficulties (Pichora-Fuller et al., 2017). This is also highly relevant with regard to work activities, since mobilizing mental resources for the listening task leads to increased listening effort and overall higher mental workload, as has been explicated in the Ease of Language Understanding (ELU) model (Rönnberg et al., 2013) and the Framework for Understanding Effortful Listening (FUEL; Pichora-Fuller et al., 2016). In other words, anything that facilitates speech comprehension frees up resources that are available for other cognitive tasks.

Contrary to what is often assumed, such age-related changes do not only occur in old age, but can already be observed from middle age onwards. For example, a recent study analyzing data from more than 300 subjects between the ages of 20 and 70 shows that under aggravated conditions, deterioration in speech comprehension already occurs from about 50 years of age (Getzmann et al., 2023). The evaluation of neurophysiological correlates of attentional control (i.e., the anterior contralateral N2 subcomponent, N2ac, and posterior alpha power lateralization) showed that these are less due to early processes of auditory search for a target stimulus and segregation of the target speaker but due to age-related changes in attentional focusing and processing of task-relevant information. While basal auditory performance did not play a significant role, more difficult auditory conditions were associated with an increased use of attentional resources, independent of the subjects’ age.

Evidence for the role of cognitive control was provided by studies on speech comprehension, which showed that especially speaker changes cause more problems for elderly people. Speaker switching is thereby viewed as a form of distraction in which a task-irrelevant feature of the speech stimulus (namely speaker and speaker position) competes with the task-relevant feature (namely speech content) for cognitive processing resources. While continuity in a speech scene facilitates speech comprehension independent of age (Mehraei et al., 2018; Meister et al., 2020), it seems to take longer to process a speaker change with increasing age. Accordingly, declines in speech comprehension immediately after a speaker change lasted longer in an older group of subjects than in younger subjects (Getzmann and Wascher, 2016). Moreover, the ability to shift attention toward task-relevant speech content was reduced in older adults as indicated by a reduced N2ac and posterior alpha power lateralization (Getzmann et al., 2020). In sum, recent findings suggest that the aging cognitive system suffers especially from the simultaneous processing of dynamic and concurrent speech content (Meister et al., 2020; Wächtler et al., 2022; Getzmann et al., 2023), which seems plausible, given that shifting, inhibiting, and updating are involved—all core processes of cognitive control proposed by Gratton et al. (2018).

There is evidence of compensation for these deficits in cognitive control of auditory attention. One form of compensation is the increased use of cues indicating an upcoming speaker switch, which are associated with increased activation in frontocentral brain areas, especially in good performers (Getzmann et al., 2017; Getzmann and Wascher, 2017). In addition, some work suggests increased use of complementary (“speech”) information by older adults, for example in the form of visual speech (e.g., Tye-Murray et al., 2016), which may act as a “fountain of youth for old ears” (Winneke and Phillips, 2011, p. 427). However, studies also demonstrated that older people do not profit *per se* more from visual language than younger people. For example, it was found that older people use visual speech stimuli less specifically to prepare for the subsequent auditory speech information (Begau et al., 2022), but they benefit more from content-congruent visual speech when integrating audio-visual speech, which—in line with the above-mentioned FUEL hypothesis—can be interpreted as a reduction of listening effort (Begau et al., 2022). Finally, and quite in accordance with the CRUNCH hypothesis, a recent review found that older adults engage additional brain regions than younger adults when performing the same cognitive task, to compensate for declining brain structures and functions (Kang et al., 2022).

### 3.2. Dual-task walking

Much like the comprehension of speech, locomotion is a crucial part of the everyday life for people of all ages. While, normally, walking does not pose a challenge itself for most, performing a secondary task while walking can be challenging, sometimes resulting in falling—with dire consequences in higher age ranges (Berg et al., 1992; Khow and Visvanathan, 2017). For a long time, the act of locomotion itself was not regarded as a task that incorporated higher cognitive processes to be executed, but newer findings have revealed cortical involvement even in imaginary walking (Jahn et al., 2008; Labriffe et al., 2017). While the integration of other tasks into the process of walking does not seem to be as challenging for younger adults, there is broad evidence from both behavioral and neuroscientific studies that older adults can face challenges while walking and executing a concurrent cognitive task (for a review, see Al-Yahya et al., 2011). With age-related decline, both sensory and cognitive functions necessary for the control of gait can deteriorate and lead to decrements, making it challenging to dual-task especially when postural stability is compromised (Woollacott and Shumway-Cook, 2002). This also poses a challenge for modern working environments, as wearable information technology or mobile phones demand the working individual to walk while maintaining engagement in a cognitive task, producing a sustained dual-task situation. During such tasks, healthy adults generally prioritize gait stability over secondary cognitive task execution, but with age-related decline in cognitive control and further cognitive impairments, concurrent information processing can lead to destabilization and falling (Yogev-Seligmann et al., 2008).

These age-related decrements in walking were not only shown on a behavioral level, but were also found in neurophysiological correlates incorporating a wide-spread network of cortical areas being responsible for many of the differences in mobility noticeable in higher age (Holtzer et al., 2014). Here, the impact of cognitive control on the allocation of resources to both the locomotor and cognitive task is crucial as it accounts for the share of the overall resources going into one of both tasks. To investigate the neurophysiological underpinnings of dual-task cognitive control mechanisms, mobile brain/body imaging techniques (MoBI) have been applied using mobile EEG and motion tracking devices. ERPs in various stages of information processing indicated key changes in electrophysiological underpinnings between younger and older adults.

A common way to investigate age effects on cognitive control is to administer an inhibitory no-go cognitive task while walking on a treadmill with self-chosen fixed speed (compared to a sedentary or standing baseline). In dual-task walking situations, a decrease in cognitive control (N2) and stimulus updating (P3) were found for older compared to younger participants while also showing higher behavioral decrements (accuracy; Malcolm et al., 2015). A high fraction of younger adults seemingly allocate cognitive resources more flexibly during dual-task walking with strong modulations in the N2 and P3 time ranges, often leading to improved performance outcomes compared to a sedentary baseline (Patelaki et al., 2023). In the same task, most older adults showed decreased accuracies while exhibiting less flexible resource allocation; though, high performing older adults demonstrated comparable behavioral and neurophysiological patterns to the younger population by maintaining a higher comparative flexibility to low-performing peers (Patelaki et al., 2023). Besides these indices for more general aging processes, an absence of higher level executive processes (indicated by the P3a component) in an inhibitory Stroop dual-task paradigm was found for elderly at risk for falling compared to healthy ones, rendering executive functions as an important factor in coordinating gait and cognition (Walshe et al., 2015).

To account for age-related cognitive decline, older adults often compensate with strategies on a behavioral level by slowing down their walking speed (Yogev-Seligmann et al., 2008), a behavior even shown in high performers (Patelaki et al., 2023). Therefore, it must be taken care of that older employees – especially those with pre-existing conditions—are not faced with a staggering amount of information processing demands while on the move.

### 3.3. Car driving

Even more than dual-task walking, safe driving can be seen as a prototypical example for the successful interaction of numerous sensory, motor and cognitive systems. Driving a car can also be seen as exemplary for the operation of machines in an occupational context. Cognitive control is particularly important when the traffic and task context is complex, for example, when the stimulus/reaction constellation relevant to driving safety has to be selected from among different stimuli and reactions, and irrelevant disruptive stimuli and inadequate reactions or competing motivational tendencies have to be suppressed.

Accident statistics worldwide and related to Germany show that older drivers (65 years and older) are not involved in more accidents overall than younger drivers (Statistisches Bundesamt, 2021). However, if the annual mileage is taken into account, which typically decreases with increasing age, the accident risk of older drivers is significantly increased and is approximately at the level of novice drivers (Ryan et al., 1998). There are many reasons for this. In addition to declining (and uncorrected) vision and motor impairments that reduce reaction accuracy and speed, it is cognitive deficits that can lead to risky driving behavior (Karthaus and Falkenstein, 2016). Accordingly, contrary to frequently expressed opinions, studies in real traffic as well as in driving simulators show that older people do not drive more unsafely *per se* than younger drivers, but often have more difficulties in conflict or critical traffic situations, e.g., intersections (Dotzauer et al., 2013). These are often characterized by a confusing number of stimuli from which the information relevant to traffic safety has to be extracted, by a high time pressure that makes it necessary to react quickly, and by the lack of adequate habitual or the presence of inadequate habitual reactions.

Experimentally, such situations can be simulated by confronting subjects during a driving task with unexpected stimuli to which they have to react quickly and adequately. In an EEG study, for example, younger and older drivers had to master a driving task in which they had to keep a vehicle in its lane and react to the brake lights of a vehicle in front (Karthaus et al., 2018a). At the same time, they were asked to respond to occasionally presented visual and auditory stimuli (but irrelevant for driving safety). It was found that especially when cognitive load was high (operationalized by a required active response to the distracting additional stimuli), older drivers’ braking response and processing of safety-relevant information deteriorated. This was evident at the neurophysiological level in the form of a reduced P3b component in the EEG, indicating an unfavorable allocation of mental resources. In a subsequent study with drivers from four age groups, a steady increase of inadequate reactions was observed, especially in the two oldest groups (i.e., from the age of 55 years), and even a dramatic increase in the age group of 70–80 years, coupled with a reduction of the P3b component (Karthaus et al., 2021). In sum, the results suggest an impaired resistance to distractor interference and a reduced inhibition of prepotent responses in older drivers especially under complex task conditions.

According to hierarchical Driver Behavior Model of Michon (1985), which distinguish between three levels of cognitive control, there are also various compensation mechanisms at work in the context of driving. Typical are forms of strategic compensation, for instance avoiding potentially risky traffic situations like driving in high traffic density and tactical compensation, e.g., reducing driving speed and maintaining a large safety distance (Michon, 1985). In addition, there are also mechanisms of situational (operational/control) compensation, which refers to direct reaction (e.g., steering, braking) and in which mental effort is adapted to the requirements of a traffic situation (Michon, 1985). An example of this was shown in a series of studies in which younger and older drivers were given the task of keeping a vehicle in its lane on a winding road for a longer period of time. Poorer performance by older drivers was associated with a more reactive response to the route guidance, which was associated with reduced alpha activity in the EEG while driving (Karthaus et al., 2018b), typically associated with states of mental withdrawal and inattention. In contrast, older drivers with good performance were characterized by proactive driving behavior and increased mental effort, coupled with low alpha activity. This suggests—in the sense of a situational compensation based on cognitive control—a higher mental workload, consumption of mental resources, and activation and sustaining of attention in good older drivers. This also fits with the results of a recent EEG driving simulation study showing that younger and also middle-aged drivers flexibly adapted their cognitive control to the difficulty of the driving task (indicated by variable theta activity in the EEG over fronto-central brain areas), whereas older drivers aged 65 years and older showed no upregulation of midfrontal theta power in response to increased steering demands (Depestele et al., 2022).

### 3.4. Work interruptions

Modern work is characterized by high demands on human information processing and EF, such as mental flexibility, or working memory. This applies in particular to the simultaneous processing of several different tasks and the management of work interruptions, which, along with time and performance pressure, are one of the greatest stress factors in the modern world of work (Rigotti, 2016). In the framework of action regulation theory (for review, Zacher and Frese, 2018) work interruptions impede action regulation and are characterized by a brief suspension of a primary task that is accompanied by the processing of a second task, after which the primary task must be resumed (Couffe and Michael, 2017; for review, Puranik et al., 2019). Work interruptions are announced by a foreseeable or unforeseeable event and are to be distinguished from disruptions and distractions which do not require immediate action. Interruptions should also be distinguished from “task switching” situations (Puranik et al., 2019). In interruptions, information about a primary task must be maintained in working memory in order to continue with the primary task after finishing the secondary task, which is not necessarily the case when switching tasks. Thus, the critical phase of shifting attention between mental representations in working memory is different in interruptions than in task switching. Overall, the demands on the cognitive system are different, making it difficult to transfer previous findings on task switching in higher age (which are quite heterogeneous according to a recent meta-analysis; Chen and Hsieh, 2023), to interruptions. However, as a similar phenomenon in both interruption and switching tasks there are so-called restart costs that are larger in older than younger adults (e.g., Enriquez-Geppert and Barceló, 2018). Interruptions typically result in degraded performance depending on the complexity of the interruption task (Cades et al., 2008; Zickerick et al., 2021), its duration (Altmann and Trafton, 2007), and timing (Botvinick and Bylsma, 2005). In addition, the ability to process interruptions appears to decline at older ages, which is typically evidenced by greater declines in performance among older individuals (Clapp et al., 2011; Clapp and Gazzaley, 2012; Arnau et al., 2019; Rösner et al., 2022).

In order to understand the nature of age-related deficits in the handling of interruptions, it is important to better understand underlying cognitive mechanisms. If there is a change from a currently pursued task to an interruption, the current processing status of the primary task and all information which will be required again later must be stored temporarily and attention must be directed to the processing of the interruption. Such a change in attentional prioritization between tasks can be studied very well based on retrospective cuing (retro-cue) paradigms. Here, information about tasks to be performed in the future is stored in working memory and attention is directed to specific working memory content by means of a cue presented during the storage phase (Griffin and Nobre, 2003; for review: Souza and Oberauer, 2016). Thus, it was found that information is stored in working memory in the form of different mental representations: Such content, which is the basis of our current actions, is stored in a prioritized state (i.e., in the focus of attention). Potentially relevant content continues to be stored in working memory and can thus also return to the focus of attention. This flexibility is central to the handling of interrupting tasks and, while some studies revealed an age-related deficit in this regard (e.g., Duarte et al., 2013; Newsome et al., 2015), others found intact focusing of attention in working memory for older adults (e.g., Gilchrist et al., 2016; Mok et al., 2016; Souza, 2016). In this respect, it can essentially be stated that the ability to focus attention in working memory does not generally decrease in old age, but especially when such processes are required for resuming a primary task after an interruption (Loaiza and Souza, 2019). This would be highly in line with the notion that cognitive deficits in older adults are specific to situations requiring attentional or inhibitory control (Hasher and Zacks, 1988).

To date, there are only few studies investigating the neurocognitive mechanisms underlying the handling of task interruptions using the EEG or other neuroimaging techniques, especially with regard to age influences. Early work used the N170 component of the ERP as a correlate of the processing of face stimuli that either had to be ignored (i.e., distractions) or responded to during a working memory task (i.e., interruptions). While the negative influence of distractions on working memory performance could be explained by a deficit in inhibiting the irrelevant face stimuli with age, a simple link between the handling of interruptions and an age-related influence on working memory functions could not be demonstrated (Clapp and Gazzaley, 2012). Additionally, a recent paper examining modulations in oscillatory theta-band EEG activity in response to a cue stimulus indicating an upcoming interruption suggested specific deficits in older adults in the cognitive control required for rapid switching between primary and interruption task goals, as well as age-related differences in reallocating cognitive resources when dealing with interruptions (Arnau et al., 2019). In a comparable way, EEG-based measures of brain oscillations have been used to investigate the relation between the difficulty of task interruptions and primary task performance: When resuming the primary task upon presentation of a visual cue, there was an increase in frontal theta power which was modulated by the prior interruption task. The lowest theta power response was evident following a cognitively high-demanding interruption task, followed by a low-demanding interruption task and a period without interruption. This has been interpreted as deficits in cognitive control of attention during resumption of the primary task after a cognitively demanding interruption task (Zickerick et al., 2021). A comparison of these processes between young and older people showed that—regardless of the difficulty of the interruption task—the ability to retain task-relevant information of the primary task in working memory and to recall it after the interruption decreased for older subjects (Rösner et al., 2022).

In summary, it can be stated that the cognitive processes underlying the handling of task interruptions can be well explained when considering the principles of shifting attention between mental representations in working memory. On this basis, the studies presented show how the cognitive-psychological mechanisms and the associated neuro-cognitive processes can be analyzed, although the influence of age-related changes has been rarely studied so far. Since the ability to handle interruptions is particularly important in the context of work environments, and especially older adults might benefit from appropriate coping strategies, it will be very important in the future to establish a closer connection between the numerous application-oriented research approaches concerning interruptions and basic experimental research on age-related impairments in attentional and working memory processes.

## 4. From the laboratory to the real life: synopsis, implications, and future directions toward a functional perspective of cognitive control

The presented examples of everyday and work-related activities have—as different as they may be—common features: They are all based on cognitive control and all show age-related changes. Accordingly, there are similar patterns in behavior (such as declining performance especially under challenging conditions but also evidence of compensation) as well as in the described electrophysiological measures. Thus, a reduction of the N2 and P3b components or an increased midfrontal theta activity are often found as correlates of an increased mental effort under high workload in older compared to younger people. Common to all examples, however, is also the distress caused by loss or limitations in these activities. Difficulties in understanding speech are described as one of the greatest limitations in old age, falling while walking under distraction is one of the most common causes of accidents among older people, the loss of a driver’s license due to declining fitness to drive is experienced as a major threats to one’s own mobility, and limitations in managing interruptions at work can result in both high strain on the individual and an increased risk of error. Cognitive control may therefore play a key role when it comes to interventions and measures to maintain and improve cognitive functioning in old age (e.g., Lustig and Jantz, 2015).

Notably, it is not only the individual who benefits, but also society as a whole. This is because each of the activities presented has a high relevance for work activities and safety. The extra effort required to maintain work performance despite age-related limitations in sensory, mental, and motor capabilities leads to a higher burden on older employees, which can increase the risk of absenteeism due to illness as well as early retirement. Given the increasing “workforce aging” (Aiyar and Ebeke, 2016), the growing labor shortage, and the claims for an extension of the working life, the improvement of key cognitive functions such as cognitive control can be seen as a target where interventions should have particularly far-reaching positive effects.

However, maintaining and improving cognitive control in old age should not be seen as an end in itself. Interventions as well as research on cognitive control could benefit considerably if more attention is paid to the functional aspect of cognitive control and its changes in everyday life of older people. Interrelationships in the field of ability—impairment—activity are highly individual and not necessarily linear. The quality of life of the individual does not necessarily increase with the improvement of his or her abilities. Possibilities for compensation and situational factors play at least as important a role (as assumed, for example, in the prominent SOC model of successful aging by Baltes and Baltes, 1990). Research on everyday cognition and aging should therefore include situational and contextual influences and conceive of individuals as intentional and functional decision makers. Practically, the study of influences of age and cognitive control would be required not only on but also in everyday activities, as well as more research in real life.

The present findings clearly show how age-related changes interact in several of the core processes of cognitive control. This involves updating, shifting, and inhibition in language comprehension in dynamic cocktail party conditions as well as in driving under complex conditions, especially shifting and inhibition in dual-task walking, and shifting and updating in dealing with work interruptions. In contrast, classical experimental tasks such as the N-back task, task switching, the Go-NoGo task, and motor control tasks mostly target single isolated processes of cognitive control. This allows for a differentiated analysis of age effects on updating, shifting, and inhibition, but less for their holistic effects on everyday activities. This is also true for compensatory mechanisms. These are partly global and independent of the specific task, such as the allocation of additional mental resources under demanding task conditions. This is reflected in comparable EEG patterns across the different activities described, for example, in the form of increased theta activity over frontal areas. In some cases, however, compensation mechanisms are inherently linked to a specific activity, such as the use of visual speech in speech comprehension, the avoidance of driving in unfavorable weather conditions as strategic compensation, or reduction of speed as situational compensation in driving. This form of compensation cannot be fully captured in classical experimental tasks.

The positive news is that for all presented examples more realistic experimental settings can be found, all benefiting strongly from the rapid development of analysis and neurophysiological methods in recent years. Hamilton and Huth (2020), for example, highlight the advantages (in addition to the challenges) of using natural language material to study language comprehension, which can be generalized to all the examples given here. Thus, to investigate cognitive processes underlying the coping with conflicting tasks in everyday life and work and to uncover the role of age-related changes, it is important to build a bridge from cognitive aging research into everyday professional practice. Frequently, this leads to the demand for more realistic research paradigms, which allow to examine the relevance for the everyday life of the aging person. The idea behind this seems to stem from the assumption that the effects found under laboratory conditions are only insufficiently transferable to everyday situations and that the findings from laboratory studies thus have little practical relevance for our lives. However, the call for more “ecological validity” so often heard in experimental cognitive research is also often accompanied by the warning for controlled conditions that allow scientifically reliable conclusions to be drawn. The examples given here demonstrate that the path from the laboratory to practice is not as long as is often assumed. There are good reasons for this: On the one hand, mobile technologies for deriving the EEG, for example, in combination with powerful analysis methods, certainly allow the reliable measurement of cognitive processes in the field (e.g., Jungnickel et al., 2019; for overviews, Gramann et al., 2017; Wascher et al., 2023). In addition, innovative statistical procedures such as modeling and decoding techniques enable the targeted testing of hypotheses in the field as well. Finally, virtual realities (VR) increasingly offer the possibility to enrich experimental environments and thus bridge the gap between laboratory and reality (for review, Wilson and Soranzo, 2015; Peeters, 2019). To do so, however, it must be ensured that VR, on the one hand, replicates reality in a sufficiently accurate way, and, on the other hand, does not place additional demands on cognition (such as when incongruent multimodal information or “impossible scenarios” are presented). Taken together, while the aim of this paper was to show examples of this, it is not intended to present simplistic cognitive neuro-scientific approaches as outdated. The review is rather intended as an impulse to use the possibilities offered by the technical and methodological developments in experimental research to take more account of the reality and everyday life of older people in the study of cognitive aging than has been the case so far.

## 5. Conclusion

The decline of cognitive control in old age is not alone of theoretical interest, but represents a serious limitation for the life and work of older people. The activities presented here make this very clear. Further development of laboratory-based study paradigms and linking the results of laboratory experiments to everyday live of older people appears to be an important, if not necessary, step in cognitive aging research in the coming years. The use of modern neurophysiological methods in the field makes this more achievable and obvious than ever before.

## Author contributions

SG and EW drafted the concept of the review article and drafted the manuscript. SG, JR, PG, MK, and DS wrote parts of the review article. All authors contributed to the article and approved the submitted version.

## Conflict of interest

The authors declare that the research was conducted in the absence of any commercial or financial relationships that could be construed as a potential conflict of interest.

## Publisher’s note

All claims expressed in this article are solely those of the authors and do not necessarily represent those of their affiliated organizations, or those of the publisher, the editors and the reviewers. Any product that may be evaluated in this article, or claim that may be made by its manufacturer, is not guaranteed or endorsed by the publisher.
